# Allostasis and sedation practices in intensive care evaluation: an observational pilot study

**DOI:** 10.1186/s40635-018-0179-0

**Published:** 2018-06-20

**Authors:** John P. R. Moore, Chris Anstey, Lauren Murray, John F. Fraser, Mervyn Singer

**Affiliations:** 1 Sunshine Coast University Hospital, Birtinya, QLD 4575 Australia; 2 The School of Medicine, The University of Queensland, 288 Herston Road, Herston, Brisbane, QLD Australia; 3 Critical Care Research Group, The Prince Charles Hospital, Rode Rd, Brisbane, QLD 4032 Australia; 4 Bloomsbury Institute of Intensive Care Medicine, Division of Medicine, University College London, Cruciform Building, Gower St, London, WC1E 6BT UK

**Keywords:** Sedatives, Critical illness, Allostasis, Multiple organ failure

## Abstract

**Background:**

A dysregulated stress response has been implicated in the pathogenesis of critical illness. Sedative agents utilised in the critically unwell patient may impact upon the stress response with a downstream negative effect on multiple organ systems. This study was designed to assess the feasibility of investigating components of the stress response as a sub-study of the current SPICE-III study (NCT01728558).

**Methods:**

This pilot observational cohort study was conducted in a single intensive care unit in Queensland, Australia. Enrolled patients were over 18 years who had been commenced on mechanical ventilation requiring sedation for less than 12 h but expected to remain ventilated for > 24 h. Blood samples were taken at 12 h intervals over a 5-day period commencing at the time of enrolment, and subsequently tested for various markers of key efferent limbs of the stress axis.

**Results:**

The 12 patients recruited closely mirrored the population within the pilot study used to design SPICE-III. Eighty-nine percent (107/120) of all planned blood samples were obtained and drawn within 0 h (0–0.3) of the planned sampling time point. Time from eligibility to enrolment was a median (IQR) 1.4 h (0.36–9.19), and time from eligibility to the first blood sample was 4.79 h (2.0–10.61). Physiological, hormonal, metabolic and cardiac biomarkers were consistent with an elevated stress response at baseline which mostly normalised over the 5-day study period. Plasma noradrenaline levels correlated with the dose of norepinephrine used.

**Conclusions:**

A larger sub-study of the SPICE-III study is feasible. The study has demonstrated a predictable trend of variation of the components of the blood panel during the evolution of critical illness and supports multiple sampling time points for the follow-up study.

**Trial registration:**

ANZCTR.org.au, ACTRN12616001200471, Registered on 22 January 2016.

## Background

Survival of an organism in the face of either internal or external events requires a measured and appropriate stress response. It has been hypothesised that an abnormal stress response is linked to the development and subsequent severity of multi-organ failure [1]. The stress response is coordinated by the primitive brain structures of the diencephalon and brainstem. It comprises a broad range of behavioural, neurohormonal, cardiovascular and other effects aimed at maintaining homeostasis. This biological system, developed over millions of years, is a critical requirement for the survival of organisms ranging from invertebrates to human beings. Conservation of this system across such a wide variety of species points to its success in priming organisms to respond to both internal and external change. In humans, stress is an indispensable part of learning and development, both physiologically and psychologically. However, when there is no escape from an inciting event, the stress response is prolonged and necessitates physiological or behavioural change in order to compensate and maintain stability. These changes themselves may generate sequelae or even complications for the organism. This is exemplified by the chronic stress-related diseases of hypertension, stroke, obesity and the metabolic syndrome [2].

Illnesses commonly treated in intensive care represent significant acute stress events. A prolonged, severe stress can transition the acute phase of critical illness into multi-organ failure. Organ failure is however postulated to preserve basic cellular survival at the expense of higher functions by inducing a metabolic shutdown. This persists until the inciting event passes and recovery is possible [3].

Critical illness may therefore represent the ultimate example of acute stress-related decompensation. Given the key role of the central nervous system, it could be predicted that sedating drugs, commonly utilised in the critically ill, will interfere with normal coordination of the stress response and thus generate abnormal outputs. If confirmed, this may contribute to the abnormal haemodynamics, metabolic disturbances and organ dysfunction observed in critical illness.

A reduction in sedation level alone confers a benefit for critically ill patients by way of reduced ventilator days, intensive care length of stay, duration of coma and mortality [4–9]. The Sedation Practices in Intensive Care Evaluation (SPICE)-III study (NCT01728558) aims to determine whether any mortality benefit can be derived from targeting light sedation utilising a protocolised sedation strategy. Pilot data from the SPICE-II study [10] show that early goal-directed sedation (EGDS) results in a marked reduction in sedation level as compared to standard practice, until approximately day 4 of admission. The SPICE-III study offers the opportunity to study two similar groups of patients who may have differing levels of physiological stress as a result of differing sedation levels (EGDS strategy versus standard care). To investigate this hypothesis, we undertook a pilot study to outline temporal changes in a panel of blood tests that represent key efferent limbs of the stress system in response to critical illness and sedation. The combination of tests was selected for the panel based on a combination of factors, namely physiological relevance, minimum total required blood sample volume and cost, in order to demonstrate feasibility and to generate baseline data to inform protocol development for a randomised controlled study.

## Methods

### Design and patients

This pilot observational cohort study was conducted in a 12-bed mixed surgical/medical general intensive care unit. The institutional human research ethics committee gave permission for prospective consent from a patient surrogate. The study was registered (ACTRN12616001200471) and conducted between August and December 2015.

Inclusion and exclusion criteria were chosen such that the study population would mirror the likely patient population in the SPICE-III study. Where relevant, data from this pilot study were compared against the standard-care arm of the SPICE-II pilot study [10] as this likely reflects the final population of the SPICE-III study. Sedation practice in the standard care arm of SPICE-II most likely reflects practice in this current study. Patients were included if they were admitted to the intensive care unit requiring invasive mechanical ventilation and likely to remain ventilated for more than 24 h. Patients were excluded if less than 18 years of age, pregnant, had been mechanically ventilated for more than 12 h, had been enrolled in the intervention arm of the SPICE-III study, or admitted with a primary cardiac diagnosis, brain lesion, drug overdose, burns, a mean arterial pressure < 50 mmHg despite adequate resuscitation, fulminant hepatic failure, were in full-time residential nursing care prior to admission, death was felt to be imminent or inevitable, or it was unlikely the patient would to survive to 90 days.

### Study outcome measures

The main outcome measures were to determine (i) baseline variability and (ii) temporal change in concentration of the components of a panel of blood tests (Table 1) taken over a period of 5 days in the context of critical illness. These data would determine the feasibility of performing blood tests on patients enrolled in the SPICE-III study in terms of recruitment rate and adherence to a protocol of blood sampling.

**Table 1:** Panel of assays performed

Aldosterone	Noradrenaline
Tri-iodothyronine (FT3)	Adrenaline
Total cholesterol	b-type natriuretic peptide (BNP)
High density lipoprotein (HDL) cholesterol	Troponin I (TNI)
Triglycerides	Ketones

### Study process

Potentially eligible participants were identified by the ICU team caring for the patient. Once informed consent was obtained either from the patient or surrogate decision maker, patients were enrolled and blood drawn immediately from an indwelling arterial catheter. Blood sampling was repeated every 12 h for a period of 5 days. The samples were processed and analysed by the local hospital pathology laboratory. FT3 was analysed using competitive binding immunoenzymatic assay, TNI by two-site immunoenzymatic (sandwich) quantitative assay, triglycerides, total and HDL cholesterol were analysed by spectrophotometric timed endpoint assay (Beckman-Coulter Synchron Clinical Systems. Brea, CA, USA). Aldosterone was analysed by liquid chromatography with mass spectrometry (LC-MS/MS). Catecholamines were analysed by high performance liquid chromatography (HPLC) coupled with electrochemical detection (ECD). β natriuretic peptide (BNP) was analysed using an iSTAT point of care analyser (Abbott Point of Care Inc. Abbott Parl, IL, USA). Ketones were analysed by FreeStyle Optimum H Blood B-Ketone Test Strips™ (Abbott, Sydney, Australia). Baseline characteristics were recorded for each patient including demographics and co-morbidities. Daily records were kept of sedation level (Richmond Agitation Sedation Scale (RASS)), the severity of illness (sepsis-related organ failure assessment (SOFA) score) and medication (type and dose of sedative, analgesic and inotropic or vasoactive agents).

### Statistical analysis

Values are reported as means with standard deviation for normally distributed variables and medians with interquartile ranges (IQRs) for non-normally distributed continuous variables and proportions for categorical variables. Comparisons between groups have been made using a chi-squared test for equal proportion or Fisher exact test where numbers were small. Where appropriate, linear regression analysis was undertaken to define the relationship between nominated predictor and outcome variables. Cross-sectional time series (panel) data were analysed using a mixed effect linear model with the patient as the panel variable. Validity of this technique was checked using a Hausman test. Significance of the regression slope (β) and its 95% confidence interval were reported. All models underwent residual diagnostics for distribution and homoscedasticity. For all analyses, the level of significance was set at *P* < 0.05.

## Results

**Fig. 1:**
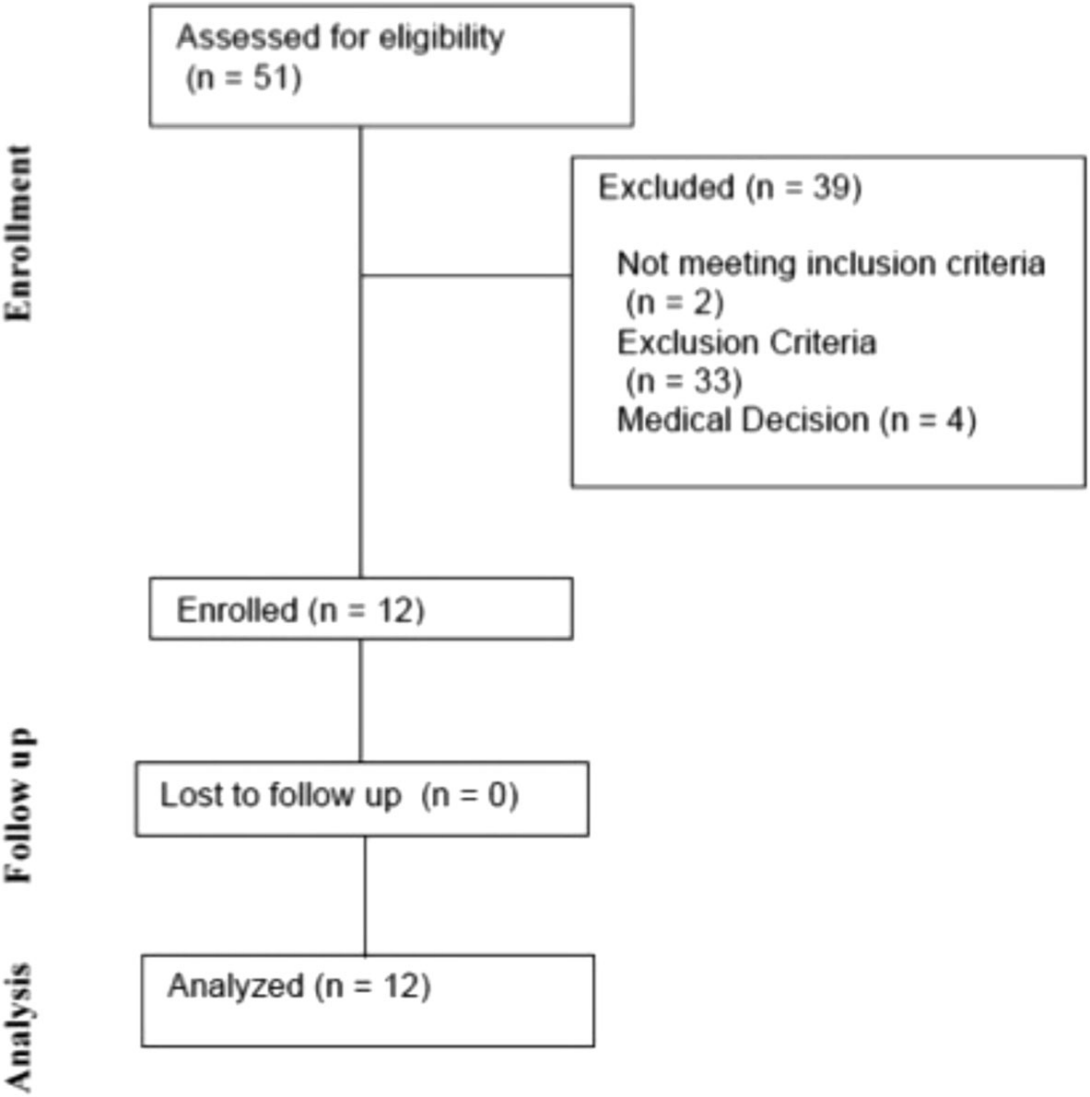
CONSORT diagram. Of the screened patients, 2 (3.9%) did not meet inclusion criteria, 33 (64.7%) met exclusion criteria and 4 (7.8%) were excluded based on medical decision

Of the 49 patients who met all inclusion criteria, 12 were enrolled (Fig. 1). Baseline patient demographics and characteristics are shown in Table 2. Patients were admitted from the emergency department (*n* = 5), general ward (*n* = 3), operating theatre (*n* = 2), medical emergency call from the ward (*n* = 1) and inter-hospital transfer (*n* = 1). There were no significant differences in baseline characteristics between this population and the standard care arm of the SPICE-II study.

**Table 2:** Patient characteristics and demographic data

Patient characteristics	SPICE-II standard arm (*n* = 16)	All-SPICE (*n* = 12)	*P* value
Age, year; mean (SD)	61.6 (17.0)	57.09 (16.30)	0.486
Male, % (*n*)	9 (56%) (9)	5 (41.67%)	0.704
Weight, Kg; mean (SD)	87.0 (28.1)	91.33 (27.68)	0.688
APACHE-II; mean (SD)	18.6 (8.8)	20.08 (10.26)	0.685
Admission source			
Ward referral, % (*n*)	25.0% (4)	33.3% (4)	0.691
Operating theatre, % (*n*)	25.0% (4)	16.9% (2)	0.673
Emergency department, % (*n*)	43.7% (7)	41.7% (5)	> 0.999
Inter-hospital transfer, % (*n*)	6.25% (1)	8.33% (1)	> 0.999
Vasopressor during study, % (*n*)	88% (14)	92% (11)	> 0.999

*APACHE II* acute physiology evaluation and chronic Health Evaluation II (11)

### Feasibility of recruitment, feasibility of blood sampling

The recruitment rate was 0.77 patients per week. No consent was revoked. Eighty-nine percent (107/120) of all planned blood samples were obtained.

The time from eligibility to enrolment was a median (IQR) of 1.4 h (0.4–9.2), and the time from eligibility to the first blood sample was 4.8 h (2.0–10.6). Initial blood samples were taken within a median (IQR) of 0.7 h (0.2–2.35) of enrolment with subsequent blood sampling done within 0.0 h (0–0.3) of the identified sampling time point (Table 3).

**Table 3:** Feasibility and clinical outcomes

Clinical outcome	SPICE-II -standard sedation limb (*n* = 16)	All-SPICE OPS (*n* = 12)	*P* value
Time from eligibility to enrolment (h), median [IQR]	1.1 [0.44–3.54]	1.4 [0.36–9.19]	*P* = 0.71
Time from eligibility to first blood draw (h), median [IQR]		4.79 [2.0–10.61]	
Time from enrolment to first blood draw (h), median [IQR]		0.655 [0.23–2.353]	
Samples obtained, % (*n*)		89.2% (107/120)	
Interval from planned to actual sample draw time (h), median (IQR)		0.0 (0–0.3)	
RASS − 2 to 1 first 48 h (%)	38% (74/197)	56% (33/59)	0.016*
RASS − 3 to − 5 first 48 h (%)	57% (112/197)	41% (24/59)	0.037*
Extubated within 7 days	75% (12)	75% (9)	> 0.999
Ventilator free days at day 28, mean (SD)	20.1 (10.1)	20.07 (9.66)	> 0.999
ICU LOS days, median [IQR]	7.0 [2.5–9.4]	6.45 [4.44]	0.471
ICU mortality, % (n)	12.5% (2)	8% (1)	0.7423
Hospital LoS, median [IQR]	17.0 [4.0–29.0]	21.15 [11.5–37.5]	0.341
Hospital mortality, % (n)	12.5 (2/16)	16.67 (2/12)	> 0.999
90-day mortality, % (n)	12.5 (2/16)	16.67 (2/12)	> 0.999

All results are presented as median [IQR]

### Physiological parameters

**Fig. 2:**
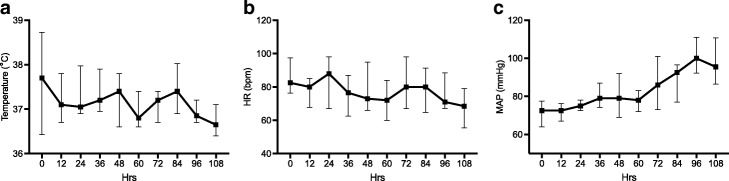
Physiological markers of stress. Median and interquartile range for each time point. **a** Temperature. **b** Heart rate. **c** Mean arterial pressure

Physiological markers of stress, namely temperature, heart rate and mean arterial pressure (MAP), are shown in Fig. 2. There was a significant drop from a median (IQR) heart rate from 82.5 bpm (76.3–97.5) to 68.5 bpm (55.5–79) (*p* = 0.023) and an increase of median (IQR) mean arterial pressure from 72.5 mmHg (64–77.5) to 95.5 mmHg (86.5–110.8). Median (IQR) core body temperature was raised at 37.7 °C at baseline with no significant change over the study period (*p* = 0.16).

### Blood results

#### Metabolic biomarkers (Fig. 3)

Total cholesterol and triglycerides showed a small but statistically significant rise over time (both *p* < 0.001). HDL cholesterol levels were subnormal at baseline and displayed no significant change over time (*p* = 0.99). There was a high degree of inter-individual baseline variability in ketone levels at baseline with a significant decrease in median level over the study period (*p* < 0.001). Only one patient received total parenteral nutrition (TPN) during the study; exclusion of this patient’s results had no significant impact on any of the above results.

**Fig. 3:**
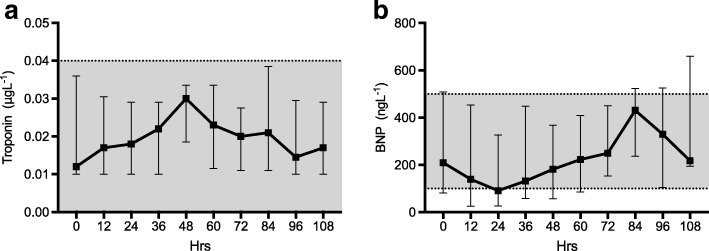
Cardiac profile. Median and interquartile range for each time point. Normal range is represented by the shaded area. **a** Troponin I, **b** β-Natriuretic peptide

Baseline median lactate levels (1.05 mmol/l [0.725–1.5]) were within the normal range and did not vary significantly (*p* = 0.38) over the course of the study. Standard base excess however increased over time (*p* < 0.001).

#### Cardiac biomarkers (Fig. 4)

In patients without a primary myocardial injury troponin I (TNI) level fell within the normal range and showed no significant change over time (*p* = 0.93). Three patients that suffered myocardial injury secondary to ischaemia, cardiac arrest and chest trauma had correspondingly elevated troponin levels. These patients were excluded from the analysis as they represent a discrete subgroup. Similarly, BNP levels fell mostly within the normal range and showed no overall trend over time (*p* = 0.79). One patient was excluded from the analysis due to a diagnosis of acute congestive cardiac failure.

**Fig. 4:**
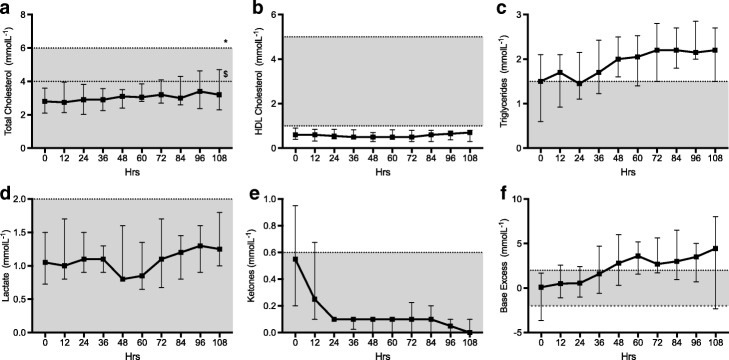
Metabolic profile. Median and interquartile range for each time point. Normal range is represented by the shaded area. **a** total cholesterol, **b** HDL cholesterol, **c** Triglycerides, **d** lactate, **e** ketones, **f** base excess. * upper limit for individuals at low cardiovascular risk. $ upper limit for individuals at high cardiovascular risk

#### Hormonal profile (Figs. 5 and 6)

Plasma adrenaline levels fell within the normal range throughout and showed no significant variation over time (*p* = 0.93). In contrast, noradrenaline levels were over tenfold higher than normal at baseline but fell by the end of the study period (*p* = 0.031). All but one patient was prescribed norepinephrine with a median [IQR] total dose of 5.2 mg [3.6–18.8] for a median of 3.5 days [2.75–7.25]. Noradrenaline levels correlated significantly with the administered noradrenaline dose (*p* < 0.001). Three patients received vasopressin. No other vasopressor or inotrope was used.

**Fig. 5:**
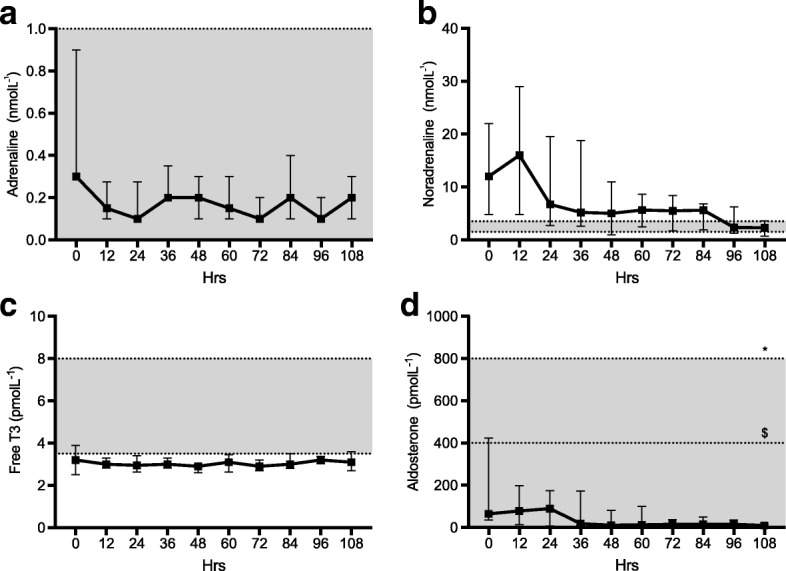
Hormone profile. Median and interquartile range for each time point. Normal range is represented by the shaded area. **a** Adrenaline. **b** Noradrenaline. **c** FT3. **d** Aldosterone

**Fig. 6:**
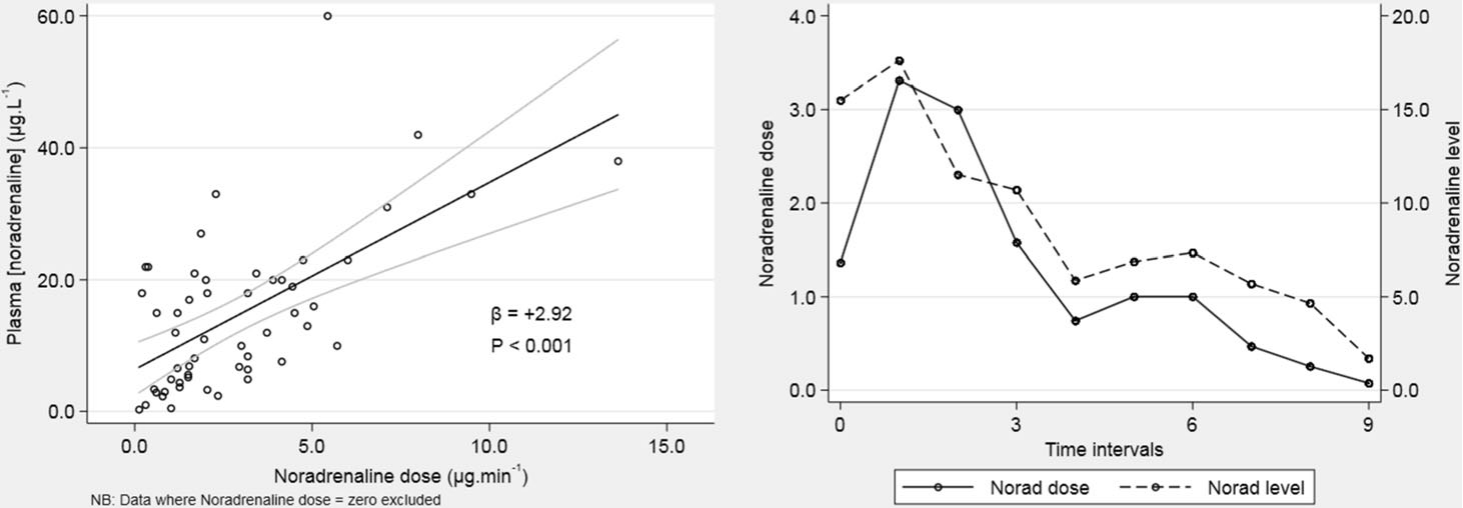
Correlation of administered noradrenaline dose (mcg/min) and plasma noradrenaline level (pmolL^−1^)

Triiodothyronine (FT3) levels were marginally low at baseline and showed no variation over time (*p* = 0.40). Aldosterone levels fell well within the normal range for supine patients but did decrease significantly over time (*p* = 0.001).

#### Sedation depth

**Fig. 7:**
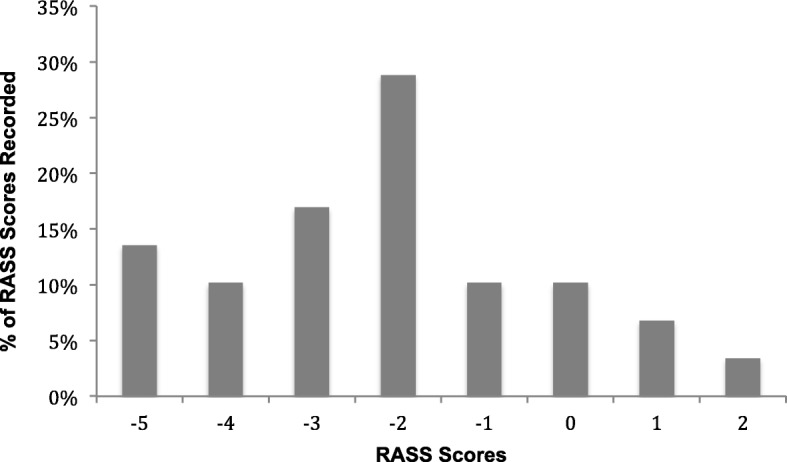
Richmond Agitation Sedation Scale (RASS) scores in the first 48 h. In the first 48 h, a total of 59 RASS scores were performed. Most of the scores were in the light sedation range (55%)

A mixture of sedating agents was used: 1 (8%) received dexmedetomidine, 10 (83%) received propofol and 10 (83%) received midazolam. For analgesia, 11 (92%) received fentanyl and 5 (42%) morphine. During the first 48 h, there was a significant difference in the level of sedation in this study as compared to the standard care arm of the SPICE-II study. Of all RASS scores, 33 of 59 (56%) were in the light sedation range versus 74 of 197 (38%) in the SPICE-II study (*p* = 0.015). On the other hand, 24 of 59 (41%) were in the deep sedation range compared to 112 of 197 (57%) in the SPICE-II standard care group (*p* = 0.03) (Table 3) (Fig 7). A similar dose of propofol (9.6 mg/kg/day [2.7–30.7]) was used as compared to that used in the SPICE-II study (11.3 [6.8–19.3] (*p* = 0.87); however, the dose of fentanyl was significantly higher (11.1 mcg/kg/day [8.1–26.2] versus 0 mcg/kg/day [0–2.7] in the SPICE-II study. There was no significant difference in the dose of morphine used as compare to the SPICE-II study (*p* = 0.576) (Table 4**)**.

**Table 4:** Cumulative dose/kg body weight and duration of treatment with sedatives and analgesic agents throughout the study period

Drugs given	Standard care *n* = 16	No. treated	All-SPICE *n* = 12	No. treated	*P* value
Dexmedetomidine (mcg/kg/day)	0 [0–0]	1	0 [0–0]	1	0.7460
Time on dexmedetomidine (day)	0 [0–0]		0 [0–0]		> 0.9999
Propofol (mg/kg/day)	11.33 [6.77–19.29]	16	9.63 [2.74–30.7]	11	0.8667
Time on propofol (day)	2.5 [2.0–4.5]		1.0 [0.5–3.0]		*0.0139* *
Midazolam (mg/kg/day)	0.037 [0–0.18]	8	0.010 [0–1.17]	10	0.466
Time on midazolam (day)	0.5 [0.0–2.0]		2.0 [1.0–4.0]		*0.0283* *
Fentanyl (mcg/kg/day)	0 [0–2.67]	7	11.14 [8.08–26.18]	11	*0.0002* *
Morphine (mg/kg/day)	0.153 [0–0.59]	10	0.20 [0–1.99]	6	0.576

Values are expressed as median [interquartile range]

## Discussion

The primary objective of this study was to demonstrate that a larger randomised sub-study of the effects of sedation upon the various axes of the stress system is both practical and feasible. This study utilised inclusion and exclusion criteria similar to that required for the SPICE-III study. The enrolment of 12 patients was completed over a 15-week period. All were recruited early within the time window of the SPICE-III study, and the delay to first blood draw was negligible. One hundred nine (90.8%) of the required 120 blood tests were performed, with loss of samples due to lack of indwelling vascular access device or discharge from the intensive care unit. These data therefore demonstrate that recruitment as a sub-study of the SPICE-III study is feasible and adherence to study protocol is likely to be excellent.

The study population closely mirrored the population from the SPICE-II pilot study that informed the design of the larger SPICE-III study. Sedation practice at the time in the participating unit utilised midazolam and propofol in preference to dexmedetomidine with a standard prescription of a RASS target range and no protocolised weaning regimen. This practice most resembled the standard care arm of the SPICE-II study. Consequently, all comparisons are made to these patients only. There was a significantly lighter level of sedation reached in our study with a correspondingly lower propofol dose. This difference likely reflects unit practice. For the follow-up study, the utilisation of multiple other sites may negate this difference.

We chose a number of variables thought likely to represent key efferent limbs of the stress axes to study changes over time in critically unwell patients following the commencement of sedation to facilitate mechanical ventilation. Each behaved differently during the observation period. Several changed significantly, reflecting a predicted pattern of stress response secondary to critical illness and its progressive resolution over time. Physiological parameters demonstrate a modest level of stress at baseline with an improvement over the time course of the study. Ketones, aldosterone, and lactate were elevated at baseline and fell to normal levels. There was also a significant drop in noradrenaline level over time; however, this is at least partially explained by reductions in the dose of administered norepinephrine. Triglyceride levels were at the upper limit of normal at study commencement and increased further during the study period. This reflects the normal response to critical illness and is a manifestation of increased lipolysis. Propofol was used in low doses only and for a short period so is unlikely to have contributed significantly to triglyceride levels. On the other hand, HDL cholesterol levels were low, a finding reflected by many other studies of critical illness [11, 12] and remained low during the time course of the study.

## Conclusion

This study has demonstrated that conduct of a larger sub-study of the SPICE-III study assessing the stress response of critical illness and modification by sedation regimen is feasible. The study has demonstrated a predictable trend of variation of the components of the blood panel during the evolution of critical illness during an intensive care admission following commencement of mechanical ventilation. In order to determine the effects of sedation level upon the components of the blood test panel, it is advisable to undertake sequential blood tests over several days.

## Abbreviations

APACHE: Acute physiology, age and chronic health evaluation
BNP: β natriuretic peptide
CONSORT: Consolidated standards of reporting trials
EGDS: Early goal-directed sedation
FT3: Triiodothyronine
HDL: High density lipoprotein
RASS: Richmond Agitation and Sedation Scale
SOFA: Sequential organ failure
SPICE: Sedation practices in intensive care evaluation
TNI: Troponin I
TPN: Total parenteral nutrition
